# Detection of Cerebrospinal Fluid Neurofilament Light Chain as a Marker for Alpha-Synucleinopathies

**DOI:** 10.3389/fnagi.2021.717930

**Published:** 2021-09-22

**Authors:** Sezgi Canaslan, Matthias Schmitz, Anna Villar-Piqué, Fabian Maass, Karin Gmitterová, Daniela Varges, Paul Lingor, Franc Llorens, Peter Hermann, Inga Zerr

**Affiliations:** ^1^Department of Neurology, University Medical Center Göttingen and the German Center for Neurodegenerative Diseases (DZNE), Göttingen, Germany; ^2^Network Center for Biomedical Research of Neurodegenerative Diseases (CIBERNED), Institute Carlos III, Madrid, Spain; ^3^Neuroscience Area, Bellvitge Biomedical Research Institute (IDIBELL), L’Hospitalet de Llobregat, Spain; ^4^Department of Neurology, University Medical Center, Göttingen, Germany; ^5^Second Department of Neurology, Comenius University, Bratislava, Slovakia; ^6^Department of Neurology, Slovak Medical University in Bratislava, Bratislava, Slovakia; ^7^Department of Neurology, Technical University of Munich, Munich, Germany

**Keywords:** alpha-synucleinopathies, biomarker, neurofilament light chain, SIMOA assay, cerebrospinal fluid

## Abstract

Alpha-synucleinopathies, such as Parkinson’s disease (PD), dementia with Lewy bodies (DLB), and multiple system atrophy (MSA), are a class of neurodegenerative diseases. A diagnosis may be challenging because clinical symptoms partially overlap, and there is currently no reliable diagnostic test available. Therefore, we aimed to identify a suitable marker protein in cerebrospinal fluid (CSF) to distinguish either between different types of alpha-synucleinopathies or between alpha-synucleinopathies and controls. In this study, the regulation of different marker protein candidates, such as alpha-synuclein (a-Syn), neurofilament light chain (NfL), glial fibrillary acidic protein (GFAP), and total tau (tau) in different types of alpha-synucleinopathies, had been analyzed by using an ultrasensitive test system called single-molecule array (SIMOA). Interestingly, we observed that CSF-NfL was significantly elevated in patients with DLB and MSA compared to patients with PD or control donors. To differentiate between groups, receiver operating characteristic (ROC) curve analysis resulted in a very good diagnostic accuracy as indicated by the area under the curve (AUC) values of 0.87–0.92 for CSF-NfL. Furthermore, we observed that GFAP and tau were slightly increased either in DLB or MSA, while a-Syn levels remained unregulated. Our study suggests NfL as a promising marker to discriminate between different types of alpha-synucleinopathies or between DLB/MSA and controls.

## Introduction

Alpha-synucleinopathies are a group of neurological disorders, including Parkinson’s disease (PD), dementia with Lewy bodies (DLB), and multiple system atrophy (MSA). They share common overriding symptoms such as rigidity, rest tremor, akinesia, autonomic, behavioral, cognitive-motor dysfunctions like freezing, and speech problems (Kompoliti and Metman, 2010). PD is the common subgroup of synucleinopathies with a 1.6% of incidence rate among people over 65 years of age (De Rijk et al., 1997). The prevalence of synucleinopathies is around 21 per 100,000 person-years, and it increases excessively with age (Savica et al., 2013). The patients with PD show clinical symptoms when approximately 50% of substantia nigra cells and striatal dopamine are lost and patients with DLB experience visual hallucinations, cognitive impairments, and conscious problems (Kompoliti and Metman, 2010).

Alpha synucleinopathies are mainly characterized by alpha-synuclein (a-Syn) aggregates in the brain tissue. While PD and DLB possess the formation of Lewy bodies (LB) in neurons (Spillantini et al., 1997), MSA is characterized by glial cytoplasmic inclusions (GCIs) in non-neuronal cells, i.e., oligodendrocytes (Wakabayashi et al., 1998).

The misdiagnosis of alpha-synucleinopathies may occur due to a clinical overlap of symptoms and the lack of specific diagnostic tests or biomarkers. A confirmed diagnosis requires postmortem examination by autopsy, which confirms approximately 80% of premortal clinical diagnoses (Savica et al., 2013).

Until present, the colorimetric ELISA system and western blotting are commonly used for protein analysis in a diagnostic context. However, small changes in the protein concentration cannot be detected effectively by these standard methods for protein analyses. The high technological progress of protein analytics had been achieved by the development of the digital ELISA concept, such as the ultrasensitive single-molecule array (SIMOA) (Rissin et al., 2010). SIMOA measures proteins in femtomolar (fM) concentrations (Rissin et al., 2010) and can detect minuscule changes in protein concentrations related to the pathological processes in the brain, which were not measurable by the standard analog methods. These alterations may be important for diagnostic and understanding of neuropathological processes.

In this study, we analyzed the concentration of potential marker protein candidates, such as neurofilament light chain (NfL), glial fibrillary acidic protein (GFAP), a-Syn, and total tau (tau) in patients with different types of alpha-synucleinopathies. Diagnostic accuracies were estimated by the receiver operating characteristic (ROC) curve analysis.

## Methods

### Patients

This study includes patients with alpha-synucleinopathies that were classified as PD (Postuma et al., 2015), DLB [criteria based on the McKeith criteria (McKeith et al., 2017)], and MSA (criteria based on the Gilman criteria) (Gilman et al., 1999, 2008); autopsies were not available (Table 1).

**TABLE 1:** Summary of the demographic information about patients and biomarker concentration.

		**DLB**	**MSA**	**PD**	**Controls**
NfL	Number of Individuals (F/M)	23 (7:16)	26 (14:12)	29 (9:20)	35 (10:22)*
	Age ± S.D.	71.17 ± 10.13	65.15 ± 11.12	66.38 ± 11.44	60.75 ± 13.39
	Meant ± S.E.M (pg/mL)	2,190.76 ± 421.46	3,839.32 ± 615.85	960.55 ± 108.82	810.00 ± 104.81

GFAP	Number of Individuals (F/M)	27 (10:17)	26 (14:12)	32 (10:22)	28 (10:15)*
	Age ± S.D.	72.03 ± 9.55	65.15 ± 12.66	65.56 ± 10.36	62.32 ± 13.49
	Meant S.E.M (pg/mL)	9,451.86 ± 1,669.36	10,291.36 ± 1,736.94	4,938.28 ± 853.62	7,693.76 ± 839.87

a-Syn	Number of Individuals (F/M)	18 (5:13)	26 (14:12)	31 (9:22)	35 (11:20)*
	Age ± S.D.	72.61 ± 9.81	65.15 ± 12.66	65.97 ± 11.01	61.06 ± 13.76
	Meant S.E.M (pg/mL)	1,468.6 ± 350.69	1,532.30 ± 336.40	1,544.61 ± 249.51	1,299.91 ± 111.32

Total Tau	Number of Individuals (F/M)	20 (6:14)	27 (14:13)	25 (9:16)	21 (5:13)*
	Age ± S.D.	71.5 ± 10.64	65.07 ± 12.43	68.32 ± 9.11	62.28 ± 12.67
	Meant S.E.M (pg/mL)	140.36 ± 27.79	80.01 ± 7.16	62.53 ± 6.21	95.51 ± 10.40

*The rest is unknown.

The control group was composed of cases diagnosed with non-primary neurodegenerative neurological and psychiatric conditions according to the acknowledged standard neurological, clinical, and para-clinical findings based on the ICD-10 definition cases, without cognitive impairment or dementia at the time of sampling. The number of patients in each group can be noted in the corresponding part in Table 1. Blood-contaminated cerebrospinal fluid (CSF) samples were excluded from the study.

### Sample Pretreatment

All protein concentrations were measured with the SIMOA-SR-X machine (Quanterix, Billerica, MA, United States). We have used commercial assay kits from Quanterix. All assays have already been optimized for certain marker proteins. In this study, we followed the protocols provided by Quanterix and applied the recommended reaction conditions to obtain the most accurate outcome. In addition, each assay contains two internal controls with a defined protein concentration. Only when both internal assay controls were in the expected range (less than 10% variation), we subjected the data for further analysis.

Kits and samples were brought to room temperature before pipetting. CSF samples were vortexed for 10–20 s and centrifuged for 5 min at 10,000 rpm to remove impurities before use.

Initially, we analyzed each sample in duplicates and observed that the intra-assay variation was marginal and not statistically significant (Supplementary Figures 1A,B). Later, we proceeded with single measurements due to low sample volumes. All samples had an identification code and were analyzed blinded and randomly by the experimenter.

### Determination of NfL

The CSF samples were diluted at 1:100 to a total volume of 100 μl. Capture antibody-coated beads (25 μl) and biotin-labeled detector antibodies (20 μl) were pipetted to the wells. Subsequently, the plate was incubated on an orbital shaker for 30 min at 30°C at 800 rpm using a black covering lid for protection from light. After washing, 100 μl of streptavidin β-galactosidase (SβG) was added to the wells, and the plates were incubated for 10 min at 30°C. After a couple of washing steps, the plate was placed in the SR-X machine for measurements.

### Determination of Tau

For tau measurements, the reaction volume was 152 μl per well. We applied the same protocol mentioned before. The dilution of CSF was 1:10. Bead and detector antibody solutions were pipetted, and the plate was incubated at 35°C at 800 rpm for 20 min on an orbital shaker. The SβG solution was prepared by mixing diluent and reagent according to the described proportion. After incubation and washing, SβG solution was added to the samples and incubated for 10 min. After several steps of washing, the plate was placed on the machine for analysis using the tau protocol.

### Determination of GFAP

We have followed the recommended dilutions according to the protocol of the manufacturer. CSF samples were diluted 1:40. Calibrators were prepared following the protocol on kit instructions. Bead solution was prepared from bead stock after washing and diluting in bead diluent. Detector reagent was also prepared according to the recommendation of the manufacturer. SβG stock solution (10 nM) was diluted to 0.150 nM. Bead (25 μl) and detector (20 μl) antibody solutions were pipetted to the wells and incubated on an orbital shaker for 30 min at 30°C with shaking at 800 rpm. After washing, 100 μl of SβG solution were pipetted to wells and incubated again at the same condition for 10 min. After SβG treatment we followed the GFAP analysis protocol.

### Determination of a-Syn

All CSF samples were diluted at 1:10 in sample dilution buffer. The preparation of bead and detector solutions was performed as described in the “Determination of GFAP” section. SβG solution was to a final concentration of 0.075 nM as the final concentration. All solutions were pipetted in the same order and the same amount as in GFAP measurement and incubated at 35°C for 35 min on an orbital shaker with shaking at 800 rpm. After washing and SβG addition, the plate was incubated for 10 min at the same condition, then the plate was transferred to the SR-X machine, and protocol for a-Syn analysis was performed.

### Statistical Analysis

The normality of values was investigated by the Dallal–Wilkinson–Lille test. For multiple comparisons, we performed non-parametric testing by the Kruskal–Wallis one-way analysis with Dunn’s test for *post hoc* comparisons. The ROC curves were calculated, and the area under the curve (AUC) values were extracted by using the software GraphPad Prism 6.0.1 (San Diego, CA, United States). All correlation studies were computed by using the non-parametric Spearman’s correlation test (two-tailed) with a CI of 95%. The *p*-values below 0.05 are considered statistically significant.

### Ethical Issues

The study was conducted according to the revised Declaration of Helsinki and Good Clinical Practice guidelines and has been approved by the Local Ethics Committee of the University Medicine Göttingen, No. 19/11/09 “Liquormarker als Prädiktoren für die Entwicklung einer Demenz bei Patienten mit Morbus Parkinson, Demenz mit Lewy Körperchen und Morbus Alzheimer” and by the Ethics Committee of the University Medicine Göttingen, No. 13/11/12 “LIX – Liquormarker zur Frühdiagnose und Krankheitsprogression bei Patienten mit Parkinson-syndromen und Motoneuronerkrankungen.”

## Results

### Determination of NfL-, GFAP-, Tau-, and a-Syn Levels in the CSF of Patients With Alpha-Synucleinopathy

The CSF samples from different subgroups of alpha-synucleinopathies (i.e., PD, DLB, and MSA) were subjected to the SIMOA analysis. The levels of four protein marker candidates, namely, NfL, GFAP, tau, and a-Syn, were measured by commercially available assay kits from Quanterix, following either a two- or a three-step protocol.

**FIGURE 1:**
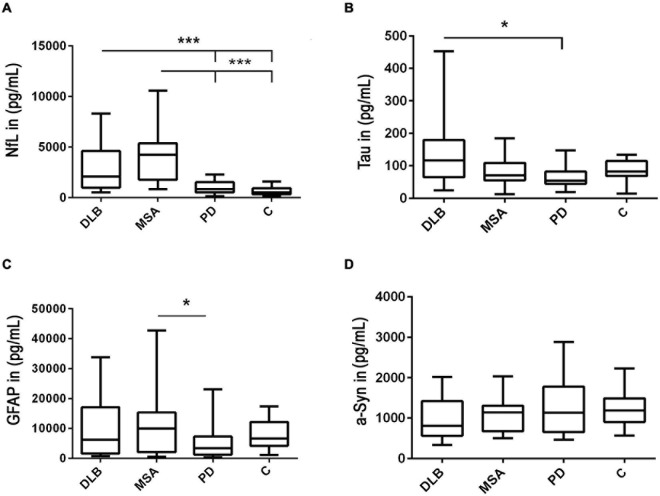
Detection of neurofilament light chain (NfL), glial fibrillary acidic protein (GFAP), alpha-synuclein (a-Syn), and tau levels in cerebrospinal fluid (CSF). Potential marker candidate proteins were determined in CSF of synucleinopathy patients with Parkinson’s disease (PD), dementia with Lewy bodies (DLB), multiple system atrophy (MSA), and controls (C). **(A)** The NfL levels were increased in patients with both MSA and DLB compared with PD and controls. **(B)** The tau levels were higher in patients with DLB than those with PD. **(C)** The GFAP levels were higher in patients with MSA than those with PD. **(D)** No significant changes in a-Syn concentrations were observed between groups. The values displayed are means and SDs. A ***p-value < 0.001 is considered as extremely significant, *p < 0.05 as significant, and *p* ≥ 0.05 as not significant.

Our measurements indicated a significant increase in the NfL levels in patients with MSA compared to those with PD and controls (*p* < 0.001). In addition, patients with DLB exhibited higher NfL concentrations than patients with PD as well as control individuals (*p* < 0.001) (Figure 1A and Table 1).

A second neurodegenerative CSF-marker, tau, was moderately increased in patients with DLB compared to those with PD and the control group (*p* < 0.05) (Figure 1B).

When we compared CSF-GFAP levels in different types of alpha-synucleinopathies, our obtained data indicated elevated GFAP levels in patients with MSA compared with PD (*p* < 0.05) (Figure 1C). In the remaining groups, CSF-GFAP levels were not significantly regulated (Figure 1C).

The levels of a-Syn, the common causative player of all synucleinopathies, were not significantly changed between all groups and it did not correlate with age (Figure 1D and Supplementary Figures 2A–C).

### Determination of Diagnostic Accuracy of NfL to Discriminate Patients With DLB and MSA From Patients With PD and Controls

**FIGURE 2:**
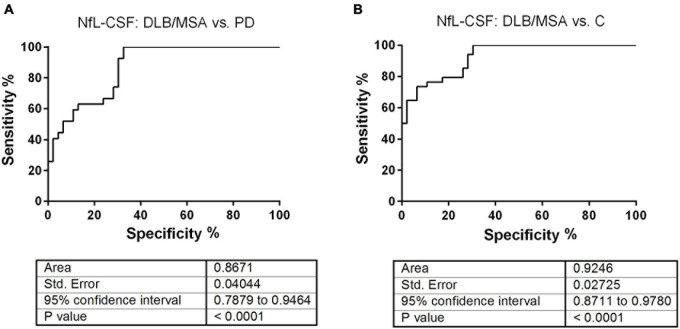
Receiver operating characteristic (ROC) curve analysis of CSF-NfL levels. To increase the statistical power, we combined DLB and MSA in one group considered as other synucleinopathies. **(A)** The diagnostic accuracy of CSF-NfL was assessed with ROC-derived area under the curve (AUC) values (with 95% CI) for the discrimination of patients with other alpha-synucleinopathies from PD. The AUC values of 0.87 ± 0.04 SD, 95% CI of 0.79–0.95 indicated a very good diagnostic accuracy. **(B)** The diagnostic accuracy of CSF-NfL was assessed for the discrimination of patients with other alpha-synucleinopathies from controls (indicated as C). The AUC values of 0.92 ± 0.03 SD, 95% CI of 0.87–0.98 indicated a very good diagnostic accuracy.

To determine the diagnostic accuracy of CSF-NfL, we conducted the ROC curve analyses using the software GraphPad Prism 6.0.1. We combined MSA and DLB to increase the statistical power. Interestingly, the ROC curve analysis indicated an AUC value of 0.87 to differentiate between DLB/MSA and PD (*p* < 0.0001) (Figure 2A). To distinguish between other synucleinopathy group and controls, our analysis revealed an AUC value of 0.92 (*p* < 0.0001) (Figure 2B). Both ROC curve analyses suggested CSF-NfL as a suitable marker to discriminate between other synucleinopathies and classical PD as well as between other synucleinopathies and controls.

**FIGURE 3:**
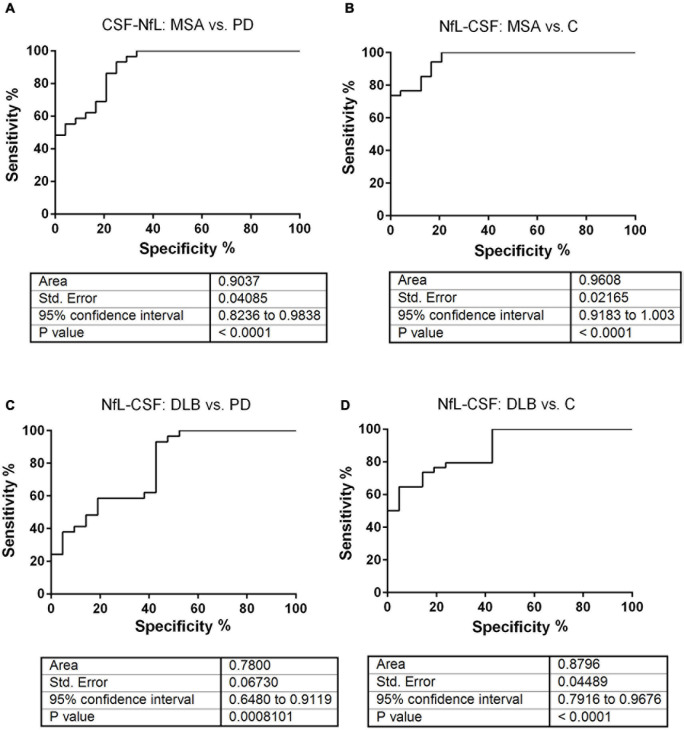
Receiver operating characteristic curve analysis of CSF-NfL levels from patients with MSA and DLB. ROC analyses were performed with the CSF-NfL concentrations obtained for the different diagnostic groups. **(A,B)** The diagnostic accuracy of CSF-NfL was assessed with ROC-derived AUC values (with 95% CI) for the discrimination of patients with MSA from PD and controls. The AUC values of 0.90 ± 0.04 SD, 95% CI of 0.82–0.98 and 0.96 ± 0.02 SD, 95% CI of 0.91–1.00 indicated a very good diagnostic accuracy. **(C,D)** The diagnostic accuracy of CSF-NfL was assessed for the discrimination of patients with DLB from PD and controls (indicated as C). The AUC values of 0.78 ± 0.07 SD, 95% CI of 0.65–0.91 and 0.88 ± 0.04 SD, 95% CI of 0.79–0.97 indicated a good diagnostic accuracy.

Subsequently, we stratified the other synucleinopathy group in MSA and DLB. The ROC curve analysis indicated that CSF-NfL differentiated MSA from PD and control cases with a very good diagnostic accuracy. The AUC values were between 0.90 and 0.96, respectively (Figures 3A,B). In contrast, the ROC curve analysis produced a lower accuracy, when we assessed the diagnostic accuracy of CSF-NfL for differentiating DLB from PD and controls, indicated by the AUC values of 0.78 and 0.88 (Figures 3C,D).

## Discussion

With populations growing and aging worldwide, the development of novel and less-invasive diagnostic test systems has become an urgent need. Currently, the diagnostic application of certain biomarkers in body fluids of patients with alpha-synucleinopathies is still a matter of debate (Eller and Williams, 2011; Schade and Mollenhauer, 2014). Therefore, our aim is to analyze the expression of potential protein marker candidates in CSF to identify a suitable marker to differentiate either between different subgroups of alpha-synucleinopathies or between other synucleinopathies and controls without neurodegeneration. We have selected four promising marker protein candidates, namely, NfL, a-Syn, tau, and GFAP, to cover a certain spectrum of neurodegeneration to inflammation. Applying a novel technology, based on SIMOA, allows us an ultrasensitive detection of these four proteins to describe potential subtle differences between groups.

### CSF-NfL as an Accurate Marker to Differentiate Between DLB/MSA and PD or Controls

In neurodegenerative diseases, CSF is a valuable source for biomarkers because it reflects neurodegenerative processes in the brain (Cramm et al., 2015, 2016; Schmitz et al., 2016a, b; Zerr et al., 2019; Llorens et al., 2020). When we determined CSF-NfL, a known marker for several neurodegenerative diseases, reflecting the neuroaxonal damage (Holmberg et al., 1998; Petzold, 2005), we obtained a highly significant increase of NfL levels in patients with DLB and MSA compared to those with PD and controls.

The ROC curve analyses suggested an accurate diagnostic accuracy of CSF-NfL to differentiate either between other synucleinopathies (i.e., DLB and MSA) from PD as indicated by an AUC value of 0.87 or between other synucleinopathies and controls as indicated by an AUC value of 0.92.

Previous studies on NfL detection in alpha-synucleinopathies already observed the elevated NfL levels in CSF of patients with MSA and DLB (Holmberg et al., 1998; Abdo et al., 2007). The diagnostic accuracy of CSF-NfL to differentiate MSA from PD was indicated by the AUC values of about 0.85–0.90 (Abdo et al., 2007; Hall et al., 2012; Magdalinou et al., 2015), which is in line with our observations. Potential differences between studies may depend on the composition of the patient cohort or the kind of methodology used for the measurement.

### Regulation of Other CSF Biomarkers in Alpha-Synucleinopathies

In addition, we analyzed the regulation of other potential biomarker candidate proteins in CSF by using assays based on the SIMOA technology. We observed a moderate tau upregulated in CSF of DLB compared to PD cases. Since we have measured tau and not the phosphorylated forms of tau in PD and DLB, which might give further information about a potential implication of tau, the diagnostic relevance of tau in synucleinopathies is limited.

The upregulation of CSF-tau in DLB is in line with our previous study (Llorens et al., 2016) using a colorimetric detection system and others (Mollenhauer et al., 2011; Mondello et al., 2014).

In addition, we observed GFAP [a type III intermediate filament and the key component of the astrocyte cytoskeleton (Eng et al., 2000; Yang and Wang, 2015)] upregulated in patients with MSA. CSF-GFAP, mainly expressed in fibrillary astrocytes, was already described to be slightly upregulated in DLB, a distinct type of other synucleinopathies, compared with controls (Ishiki et al., 2016).

We observed no significant alterations of CSF-a-Syn levels between different diagnostic groups. Previous studies using a colorimetric a-Syn assay or an assay based on the Mesoscale technology revealed a moderated decrease of CSF-a-Syn in alpha-synucleinopathies compared with controls. No significant regulation was reported between different subgroups of synucleinopathies, such as PD, DLB, and MSA, which is in line with our current study (Llorens et al., 2017; Schmitz et al., 2019).

The strength of our study is that we applied the novel and ultrasensitive SIMOA to measure potential CSF protein marker candidates for alpha-synucleinopathies. The assays are commercially available and well-validated. In future studies, we also plan to develop a Homebrew assay for the detection of epigenetically modified a-Syn or tau species, such as different phosphorylated forms. A limitation of this analysis is the low number of available DLB cases that impeded a proper calculation of diagnostic accuracy. Therefore, a confirmation of our observations in larger cohorts or with alternative methodologies (e.g., analog or multiplexing detection systems) is suggested.

## Conclusion

Our study applied an innovative digital methodology (i.e., SIMOA), which enabled us to detect even subtle regulations in CSF, suggesting NfL as a potential diagnostic marker for a reliable discrimination either between other synucleinopathies (i.e., DLB and MSA) and PD or between other synucleinopathies and controls. This is of particular interest because, in the daily clinical routine, it is more difficult to differentiate the PD-type of MSA (MSA-P) and PD than to differentiate MSA and DLB. Therefore, NfL may become important as a marker to identify those patients, allowing to include them in disease-modifying trials early in the disease course and to inform them about the quite different prognosis and characteristics of MSA disease.

## Data Availability Statement

The raw data supporting the conclusions of this article will be made available by the authors, on request.

## Ethics Statement

The study was conducted according to the revised Declaration of Helsinki and Good Clinical Practice guidelines and has been approved by the Local Ethics Committee of the University Medicine Göttingen, No. 19/11/09 “Liquormarker als Prädiktoren für die Entwicklung einer Demenz bei Patienten mit Morbus Parkinson, Demenz mit Lewy Körperchen und Morbus Alzheimer” and by the Ethics Committee of the University Medicine Göttingen, No. 13/11/12 “LIX – Liquormarker zur Frühdiagnose und Krankheitsprogression bei Patienten mit Parkinson-syndromen und Motoneuronerkrankungen.” The patients/participants provided their written informed consent to participate in this study.

## Author Contributions

MS and IZ designed the study and wrote the manuscript. SC performed the experiments. SC, MS, AV-P, and PH analyzed and interpreted the data. AV-P, KG, PL, PH, FM, and FL critically revised the manuscript. KG, FM, and DV provided the samples and the clinical data. All authors contributed to the article and approved the submitted version.

## Conflict of Interest

The authors declare that the research was conducted in the absence of any commercial or financial relationships that could be construed as a potential conflict of interest.

## Publisher’s Note

All claims expressed in this article are solely those of the authors and do not necessarily represent those of their affiliated organizations, or those of the publisher, the editors and the reviewers. Any product that may be evaluated in this article, or claim that may be made by its manufacturer, is not guaranteed or endorsed by the publisher.

## Supplementary Material

Supplementary Figure 1 Analysis of intra-assay variations of NfL in CSF. We chose different concentrations (high and low) to compare pairwise the NfL levels measured in duplicates on the same plate **(A)**. Statistical analysis revealed no significant (ns) between both measurements **(B)**, *p* > 0.05.

Supplementary Figure 2 Analysis of a potential correlation between a-Syn levels and age. **(A–C)** In synucleinopathy patients and controls, we correlated aSyn levels and age. No correlation was detected between all groups. All correlation studies were computed by using the non-parametric Spearman’s correlation test (two-tailed) in a CI of 95%. A ^∗^*p*-value < 0.05 was considered as significant and *p* ≥ 0.05 as not significant.

## Abbreviations

a-Syn: alpha-synuclein
AUC: area under the curve
CSF: cerebrospinal fluid
GFAP: glial fibrillary acidic protein
DLB: dementia with Lewy bodies
LB: Lewy bodies
MSA: multiple system atrophy
NfL: neurofilament light chain
PD: Parkinson’s disease
ROC: receiver operating characteristic
SIMOA: single-molecule array
tau: total tau.
