# Supplementary figures and images for: Detection of Cerebrospinal Fluid Neurofilament Light Chain as a Marker for Alpha-Synucleinopathies
Source: Front Aging Neurosci. 2021 Sep 22;13:717930. doi: 10.3389/fnagi.2021.717930 (PMC8493247; doi:10.3389/fnagi.2021.717930)

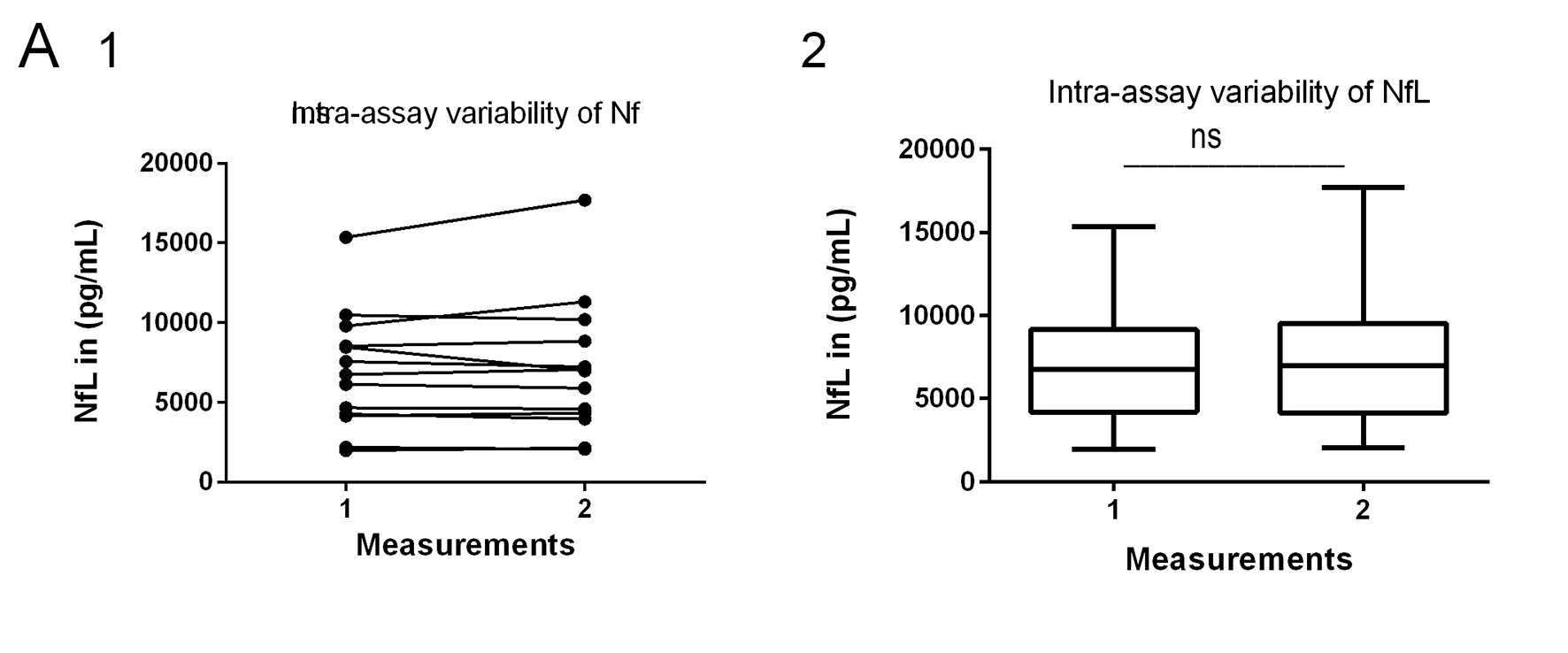

Supplement: Supplementary Figure 1 — Analysis of intra-assay variations of NfL in CSF. We chose different concentrations (high and low) to compare pairwise the NfL levels measured in duplicates on the same plate (A). Statistical analysis revealed no significant (ns) between both measurements (B), p > 0.05. [file Image_1.TIF]

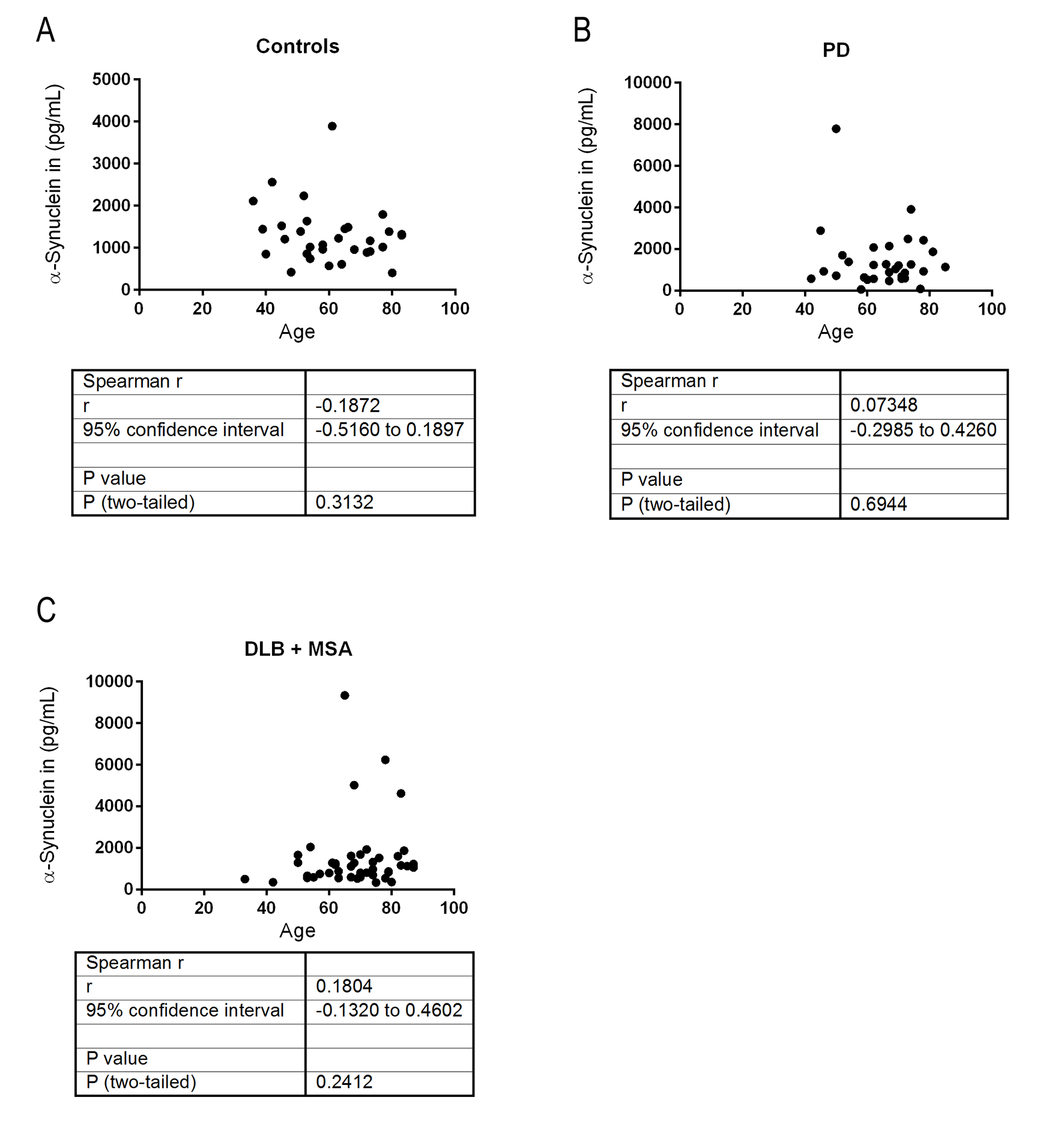

Supplement: Supplementary Figure 2 — Analysis of a potential correlation between a-Syn levels and age. (A–C) In synucleinopathy patients and controls, we correlated aSyn levels and age. No correlation was detected between all groups. All correlation studies were computed by using the non-parametric Spearman’s correlation test (two-tailed) in a CI of 95%. A ∗p-value < 0.05 was considered as significant and p ≥ 0.05 as not significant. [file Image_2.TIF]
